# Biorelevant *In Vitro* Release Testing and *In Vivo* Study of Extended-Release Niacin Hydrophilic Matrix Tablets

**DOI:** 10.1208/s12249-019-1600-z

**Published:** 2020-01-27

**Authors:** Bartłomiej Milanowski, Arkadiusz Hejduk, Marek A. Bawiec, Emilia Jakubowska, Agnieszka Urbańska, Anna Wiśniewska, Grzegorz Garbacz, Janina Lulek

**Affiliations:** 1 Department of Pharmaceutical Technology, Faculty of Pharmacy, Poznan University of Medical Sciences, 6 Grunwaldzka Str., 60-780 Poznan, Poland; 2 LEK-AM Pharmaceutical Company Ltd., 14A Ostrzykowizna Str., 05-170 Zakroczym, Poland; 3 Faculty of Electronics, Department of Computer Engineering, Wrocław University of Science and Technology, 27 Wybrzeze Wyspianskiego Str., 50-370 Wroclaw, Poland; 4 Physiolution GmbH, Walther-Rathenau-Strasse 49a, 17487 Greifswald, Germany; 5 Physiolution Polska Sp. z o.o., 74 Pilsudskiego Str., 53-025 Wroclaw, Poland

**Keywords:** niacin (nicotinic acid), extended-release tablets, *in vitro* release studies, biorelevant dynamic conditions, pharmacokinetics, bioequivalence

## Abstract

Niacin (nicotinic acid, NA) is administered orally as an antihyperlipidemic agent in extended-release (ER) tablets in high doses. Due to rapid absorption and extensive metabolism (non-linear pharmacokinetics), the drug plasma levels are highly variable, which may correlate with side effects. Interestingly, this erratic drug delivery behavior of niacin ER products cannot be clarified by compendial *in vitro* release testing. The standard dissolution tests do not allow to mimic the selected GI tract characteristics in order to estimate the robustness of formulation under the variability of the physiological conditions. These are characterized by the pH value, impact of motility forces and composition, as well as volume of GI liquids. Our paper demonstrates a comparison of a newly developed ER HPMC niacin formulation with an originator product. The research aimed to design a robust matrix tablet of comparable biopharmaceutical behavior, safety and efficacy. The extensive *in vitro* investigation, including dynamic studies in flow-through cell apparatus and stress test device, forms the basis for the evaluation of nicotinic acid plasma concentrations *in vivo*. The occurrence of erratic, multiple NA plasma peaks after the administration of both extended-release products is a result of its local input excess over the metabolic threshold (at the level corresponding to maximum 2% of the administered dose, i.e., 20 mg of drug) due to the mechanical stresses of physiological intensity. We demonstrate how this behavior is similar for both marketed and test products. In this context, we describe how a robust ER matrix and well-designed formulation does not guarantee the test product’s bioequivalence to the comparator one out of reasons unrelated to technology and biopharmaceutical properties, but because of the active compound’s intrinsic pharmacokinetic characteristics, i.e*.*, highly variable, extensive metabolism of nicotinic acid.

**Electronic supplementary material:**

The online version of this article (10.1208/s12249-019-1600-z) contains supplementary material, which is available to authorized users.

## INTRODUCTION

Niacin (nicotinic acid) is the oldest drug used for the treatment of dyslipidemias, known since the 1950s (1). It displays a broad mechanism of action, affecting favorably all of the components of blood lipid profile which are the markers for atherosclerosis and cardiovascular (CV) disease risk. Niacin reduces the level of proatherogenic low-density and very low-density lipoprotein cholesterol (LDL-C and VLDL-C), as well as plasma triglycerides (TG). Simultaneously, it markedly raises high-density lipoprotein cholesterol (HDL-C), the fraction of lipoproteins considered to exert atheroprotective action (2).

As a Biopharmaceutics Classification System (BCS) class I drug (highly soluble, highly permeable), nicotinic acid is a typical candidate for modified release formulation. With a pKa value of 4.84 (3) and fast dissolution, it is readily absorbed along the GI tract. For niacin concentration ranges relevant to its pharmacological doses, active uptake has been postulated via non-specific, pH-dependent carriers with affinity to monocarboxylic acids, including proton cotransporter and bicarbonate antiporter mechanisms (3,4). Moreover, pH-dependent, saturable nicotinic acid uptake transporter was found in human colonocytes (5). The possibility of niacin absorption in the large intestine serves as the rationale for designing extended or sustained release formulations.

The most common adverse effect reported for niacin is skin flushing and pruritus (6). Over the years, it was observed that niacin formulations with different drug release rates exhibit different occurrence rates and severities of dermal reactions, i.e., the highest one for immediate-release (IR) niacin (complete drug absorption within 1–2 h), the lowest for so-called sustained release (SR) niacin (absorption in over 12 h) (7). Initially, this phenomenon was attributed to different metabolite formation according to two distinct pathways. The glycine conjugation path results in the formation of nicotinuric acid (NUA) and is characterized by low substrate affinity and high capacity. The other pathway, of high affinity and low capacity, leads to nicotinic acid amidation to nicotinamide (NAM) and several of its secondary metabolites (7). The proportion of metabolites formed after nicotinic acid administration was shown to be governed by the rate of its absorption, with marked differences between immediate and sustained (“timed”) release formulations (8,9). With slow release and absorption, the predominant metabolic course is the amidation pathway. Due to its low capacity, faster rates of niacin input cause its saturation, which in turn directs the excess of nicotinic acid to conjugation pathway. Therefore, the predominant metabolite formed after the administration of IR niacin products is nicotinuric acid, while after SR formulations - various products of nicotinamide biotransformation were formed.

For a long time, it was postulated that this phenomenon is responsible for different adverse events profiles; with NUA inducing flushing and pyrimidine intermediates causing hepatotoxicity observed in some SR formulations, a belief still present in relatively recent reviews as well (10). However, this view was challenged with the discovery of GPR109A receptors for nicotinic acid in the skin, whose activation triggers the release of prostaglandin D_2_. Therefore, vasodilatation and related dermal manifestations are caused by unmetabolized nicotinic acid, not NUA (11). In the light of these statements, the increased incidence of flushing with immediate-release niacin products is explained by the appearance of multiple local peaks of free nicotinic acid in plasma, when its release and absorption rate exceeds the threshold capacities of both metabolic pathways (12).

Based on the aforementioned considerations, the formulation of choice in contemporary niacin pharmacotherapy is the extended-release (ER) formulation, originally designed to mitigate dermal flushing frequency and severity on the one hand and to avoid potential hepatotoxic metabolic pathway on the other hand, owing to its intermediate nicotinic acid release and absorption rate (8–12 h) and favorably balanced metabolic profile (7,10). ER niacin products exhibit good safety characteristics with little to no serious adverse effects (13) and are significantly and remarkably safer than other types of formulations (14).

A major characteristic of niacin pharmacokinetics is its vast interindividual variability due to extensive metabolism. Variation coefficients (%CV) of c_max_ and AUC values for nicotinic acid plasma concentrations have been reported in different ranges, depending on the particular study, e.g., 70–105% (15) to even 123–147% (16). The degree of variability is related to niacin dosing rate, with faster input resulting in reduced %CV (9). Moreover, sex differences have been observed in Chinese population, with females achieving generally higher systemic exposures to nicotinic acid (NA) and nicotinuric acid (NUA), as well as displaying multiple plasma NA and NUA peaks of unknown cause (17).

Due to extensive metabolism with product proportion dependent on administration rate/release kinetics, the extent of niacin’s absorption, as well as absolute and relative bioavailability of its oral formulations, cannot be reliably assessed by measuring only the plasma concentrations of the parent compound. Instead, it is recommended to calculate the total urinary excretion of nicotinic acid and its major metabolites: NUA, methyl-nicotinamide (MNA) and N-methyl-2-pyridone-5-carboxamide (2PY), which typically indicate the elimination of over 70% of the administered dose within 72 h (9,18). This fact, taken together with very high interindividual variability, makes it especially difficult to compare different formulations or treatments *in vivo* and assess their bioequivalence, as shown by Kosoglou et al. (16).

Extended-release formulations of BCS class I drugs are considered as typical products for developing *in vitro*–*in vivo* correlations (IVIVC), mathematical models that enable the predictions of a particular product’s *in vivo* absorption or plasma drug concentration after its administration based on the results of its *in vitro* release testing by an appropriate biorelevant method (19). Since highly soluble, highly permeable drugs such as nicotinic acid are readily dissolved and almost instantly absorbed in the intestine, the rate-limiting step for their absorption process from ER formulations is controlled by the product properties - release kinetics. However, niacin’s systemic availability is heavily influenced by extensive first-pass metabolism, which is also the source of major variability in NA plasma concentrations. In such cases, the development of IVIVC is difficult to achieve and not recommended, as plasma drug concentration predictions would inevitably be confounded by the active’s intrinsic pharmacokinetic properties (20).

Nevertheless, attempts at IVIVC for extended-release niacin have been described. Turner et al. developed two controlled release matrix formulations (HPMC- and PEO-based, respectively) and compared it with the commercial product (21). The release kinetics was found to be determined by the matrices’ swelling and erosion, and at least a qualitative relationship between *in vitro* release profiles (USP apparatus 2, phosphate buffer pH 6.8) and plasma nicotinic acid AUC values was established. The authors also subjected their tablets to texture analysis in order to check their resistance to mechanical stress corresponding to gastrointestinal contraction forces (21). An ER matrix must be sufficiently strong to prevent burst release events throughout the GI passage, which in the case of niacin products would result in sudden spikes and additional peaks of NA plasma concentration potentially leading to flushing incidents. Therefore, simulating and assessing mechanical resistance to GI stress is crucial for developing a high-quality ER niacin formulation, as we demonstrate in the following paper. While Turner et al. claim to have achieved a level A IVIVC, the actual study design points to a qualitative, not quantitative, *in vitro*–*in vivo* correlation (21).

On the other hand, Kesisoglou et al. developed a quantitative, multiple level C correlation for ER niacin combination product with laropiprant, based on three formulations with varying HPMC levels as a matrix former (22). However, the group did not establish a level A correlation, usually expected with successful multiple level C development, which was caused by the drug’s extensive metabolism and high variability, accounted for by sufficient for IVIVC design sample size (36 subjects). In the authors’ words, “the difficulties to establish the IVIVC cannot be attributed to the study or formulation selection, rather this represents an inherent limitation of the compound pharmacokinetic properties” (22).

The paper presented here demonstrates a comparison of a newly developed extended-release niacin formulation with an originator product. The aim of the research was to design a robust matrix tablet of comparable biopharmaceutical behavior, safety, and efficacy. The extensive *in vitro* investigation (including dynamic studies in flow-through cell apparatus and stress test device) forms the basis for the evaluation of nicotinic acid plasma concentrations *in vivo* and the occurrence of erratic, multiple NA plasma peaks after the administration of both extended-release products. We demonstrate how this behavior is similar for both marketed and test product. To the authors’ knowledge, the presented work is the first in the publicly available literature to comprehensively describe a successful reduction in product-related variability for a test formulation against a comparator for extended-release niacin, backed by different approaches to biorelevant *in vitro* dissolution testing.

## MATERIALS AND METHODS

### Reagents

Niacin USP granular special lots 313 and 515 with purity 99.9% and 100.15%, respectively, were purchased from Lonza Ltd.; methanol-gradient grade and formic acid (analytical grade) from Merck and dissolution media concentrates of 0.05 M acetate buffer solution pH 4.5 and 0.05 M phosphate buffer solution pH 6.8 were purchased from JTBaker; sodium chloride, calcium chloride, potassium chloride, magnesium sulfate, sodium dihydrogen phosphate anhydrous, sodium hydrogen carbonate, sodium dihydrogen phosphate dehydrate, sodium hydroxide microgranulates, concentrated hydrochloric acid 35–38%, phosphoric acid 85%, glacial acetic acid 99.5–99.9%, and ethyl alcohol 96% were used at analytical grade and acquired from AppliChem, Avantor Performance Materials S.A., Sigma-Aldrich, and VWR Chemical; Tween 80 was purchased from ACROS Organics. Cannula filters PSFIL001-EW-1000 with 1-μm diameter pore size were purchased from Erweka GmbH, while MGB-grade glass-microfibre discs were obtained from Munktell-Filtrak. SIF Powder Original used for fasted- or fed-state simulated intestinal fluid (FaSSIF and FeSSIF) was received from https://biorelevant.com/. Water was purified in the Millipore water purification system.

### Investigated medicinal products (IMPs)

In our study, we used the following comparator (reference) products: Niaspan^®^, niacin extended-release tablets 1000 mg, batches no. 1008542 (encoded as Ref_1, AbbVie Inc., USA) and no. 05260AF (encoded as Ref_2, Abbott Laboratories, USA), as well as test products nicotinic acid LEK-AM 1000 mg, extended-release tablets, batches no. N100010715.1 (encoded as Test_1) and no. N100020715.1 (encoded as Test_2), LEK-AM Pharmaceutical Company Ltd., Poland). The reference products were purchased from US market, whereas test products were manufactured in LEK-AM standard production area as pilot production batches. The composition of the test products tablets was nicotinic acid USP (Lonza) – 80.11% w/w and hypromellose (HPMC, Dow Chemical Company), povidone (PVP, Ashland), and Opadry II coating system (Colorcon) in appropriate proportions, as well as purified water – q.s.. Direct compression (DC) technology was used to obtain LEK-AM matrix tablets, i.e., the drug was blended with HPMC and PVP in a bin tumbler for 20 min and sized through the sieving machine fitted with a 1.5 mm screen. The final blend was fed into a hopper, and caplet-shaped tablets were compressed using biconcave oblong shape punches with dimensions 19.0 × 9.6 mm and die on a rotary tablet press (Kilian Pressima). The hardness of the obtained tablet cores was in the range 160–240 N. The tablet cores were film-coated in a perforated drum (Unitypharm D50/15 coater). The coated tablets were packaged into PVC/PVdC90-g/m^2^ transparent foil sealed with aluminum lidding foil.

### Standard *in vitro* release test conditions

*In vitro* release tests were performed on paddle apparatus (Ph. Eur. apparatus 2 equipped with basket sinkers, DT 1420 Erweka, Heusenstamm, Germany) at 37 ± 0.5°C, 50 rpm, 900 mL fill volume, sampling time points: 1, 3, 6, 9, 12, 16, 20, and 24 h. Standard compendial dissolution media were used, i.e., 0.1 M HCl pH 1.2, 0.05 M acetate buffer pH 4.5, 0.05 M phosphate buffer pH 6.8, and purified water (quality control medium recommended by FDA). After equilibration of medium temperature in the range of 37.0 ± 0.5°C, one tablet was placed into basket sinker in each vessel, and analysis was performed according to established parameters. After the specific time, 5 mL of solution was withdrawn from each vessel and filtered with 0.2-μm PVDF syringe filter. The first portion of the filtrate was rejected. The solution was made up to the volume with the same medium in the temperature range of 37.0 ± 0.5°C. Release test was performed for 12 tablets of each product in 4 selected media. Drug concentrations were determined using validated HPLC-UV method at 250 nm using Zorbax SB-CN 3.5 μm, 4.6 × 150 mm chromatographic column and methanol/formic acid/H_2_O mixture (20:2:1978 v/v/v) as mobile phase with 1.0 mL/min flow rate, injection volume was 5.0 μL, and the temperature of the autosampler and HPLC oven were 20°C and 30°C, respectively. The percent [%] of drug released in each sampling time point was calculated.

### Biorelevant fasted state conditions using flow-through cell apparatus

The release processes were also investigated in an open-loop semi-automated flow-through cell dissolution system (SOTAX AG, Allschwil, Switzerland) using the method defined by Chapter 2.9.3 Dissolution (apparatus 4) described in Ph. Eur.. The dissolution system consisted of SOTAX CE 7 smart unit with a set of seven cells with 22.6 mm diameter for large tablets, SOTAX CP 7–35 piston pump, four position media selector SOTAX MS 47, reservoirs for the dissolution medium, and double-beam UV-Vis spectrophotometer Nicolet Evolution 300 (Thermo Electron Corporation, USA). Each cell for tablets was prepared by placing a 5-mm ruby bead in the apex of the cone, and the lower conical part of the cell was filled with 1-mm glass beads. Tablets were placed on holders. The cell was closed with prepared insert and filter head containing one glass microfiber filter (Munktell^®^ MGB with retention capacity of 1.0 μm). The open-loop configuration used a sequence of biorelevant dissolution media simulating intestinal conditions for the fasting state with physiological media gradients and transit times, i.e., the stomach: simulated gastric fluid sine pepsin (SGFsp) pH 1.8 + 0.1% Tween 80 for 1 h; small intestine, blank FaSSIF pH 6.5 + 0.1% Tween 80 for 4 h; and colon, simulated colonic fluid (SCoF) pH 5.8 + 0.1% Tween 80 for 19 h. Media reservoirs were connected to the pump via media selector. The medium was pumped through each flow cell with a constant flow rate of 4 mL/min maintained at ± 1% (this is the lowest flow rate recommended by Ph. Eur. and USP). The eluents were filtered within cell heads and transferred directly to the spectrometer for analysis. On-line UV measurements at 262 nm were taken in flow-through cuvettes with 1-mm path length (Starna Scientific Ltd., Hainault, United Kingdom) at predetermined times. The WinSOTAX Plus Dissolution Software automatically read the baseline for each cell, recorded raw absorbance data, corrected data, and calculated concentration as well as % of the released drug. Detailed release test conditions are listed in Table SI in Supplementary Materials.

### Dynamic release stress test method under simulated fasted and fed conditions

The release stress tests under simulated fasted and fed conditions were performed using the device described by G. Garbacz and W. Weitschies (23) and presented in Supplementary Materials. The dissolution stress test apparatus was operated using one test program intended for simulation of fasting and one program intended for simulation of fed intake conditions of bioequivalence studies. The arrangement of the test program used for the simulation is summarized in Tables I and II.

**Table I:** The test program and the media change pattern used in the dissolution stress test device intended for simulation of fasting intake conditions

Program 1			
Intragastric condition	Gastric emptying	Intestinal passage	Ileocaecal and colonic passage
0–1 h No agitation	1 h 3 pressure waves of 300 mbar fortitude + 1 min rotation at 100 rpm	1–5 h No agitation	5, 9, 12, 16, and 20 h 3 pressure waves of 300 mbar fortitude +1 min rotation at 50 rpm
Medium: SGFsp pH 1.8		Medium: 1 mM Hanks hydrogen carbonate buffer pH 6.8 SIF-powder in FaSSIF-concentration and 5 mM KH_2_PO_4_

**Table II:** The test program and the media change pattern used in the dissolution stress test device intended for simulation of fed intake conditions

Program 2			
Intragastric conditions	Gastric emptying	Intestinal passage	Ileocaecal and colonic passage
2 h, 3 h, 4 h 2 pressure waves of 150 mbar fortitude + 0.5 min rotation at 50 rpm	5 h 3 pressure waves of 300 mbar fortitude + 1 min rotation at 100 rpm	5–9 h No agitation	9, 12, 16, and 20 h 3 pressure waves of 300 mbar fortitude + 1 min rotation at 50 rpm
Medium: 50 mM phosphate buffer (KH_2_PO_4_) pH = 5.5 for 0–1 h pH = 4.5 for 1–2 h pH = 3.0 for 2–5 h		Medium: 50 mM phosphate buffer (KH_2_PO_4_) with addition of SIF-powder in FeSSIF concentration pH = 6.8	

Samples of dissolution media were filtered during sampling through a 1-μm Poroplast-filter (Erweka Heusenstamm, Germany) and measured without further pre-treating. Amount of the dissolved drug was determined using UV–Vis spectroscopy (Agilent 8453, Agilent Technologies, Santa Clara, USA) in the closed-loop configuration. The absorbance was measured every 10 min using quartz flow-through cells (Hellma, Müllheim, Germany) equipped within 1-mm light path in differential mode at 262 (signal) and 295 nm (background).

The method was characterized by simplicity, reproducibility, and robustness toward tablet excipients and changes in the media composition. These features make it useful for routine application for the determination of the amount of the drug dissolved in simple and biorelevant dissolution media.

### Statistical evaluation of release data

Exploratory data analyses were performed using DDSolver (24). Unless stated otherwise, results (as the mean ± SD) are presented in plots as fraction released [%] in appropriate dissolution media and plasma concentrations [ng/mL] *vs.* time [h]. Release profiles similarity was determined using the f_2_ statistic (according to Guideline CPMP/EW/QWP/1401/98 Rev.1/Corr** “Guideline on the Investigation of Bioavailability and Bioequivalence”) (25). Release profiles were similar if f_2_ > 50.

### Clinical evaluation of investigated medicinal products (*in vivo* study in humans)

In the pilot study trial no. 134–13, the bioequivalence (BE) of sponsor’s test formulation in comparison to the reference formulation was assessed. It was an open-label (laboratory blinded), balanced, randomized, two-treatment, two-sequence, four-period, single oral dose, crossover bioequivalence study in 14 healthy, adult, human subjects under fasting and fed conditions.

In the first two periods (periods I and II), after an overnight fast for at least 10 h, subjects were administered, the IMPs in sitting posture with 240 ± 2 mL of drinking water at ambient temperature. In the last two periods (periods III and IV), after fasting overnight for at least 10 h, the subjects were served with US FDA recommended high-fat and high-calorie vegetarian breakfast, which they were required to consume completely within 30 min. The investigational product was administered to the subjects in sitting posture with 240 ± 2 mL of drinking water at 30 ± 2 min after breakfast. Lunch was provided 5 h after dosing. After lunch, the standardized meal was served to the subjects at appropriate times (i.e., snack after 8 h and dinner after 11 h). Subjects were provided nicotinic acid controlled diet throughout their stay within the clinical facility. A total of 18 venous blood samples were collected from each subject in all periods at 0- (pre-dose), 0.25-, 0.5-, 0.75-, 1.0-, 1.25-, 1.5-, 2.0-, 2.5-, 3.0-, 4.0-, 5.0-, 6.0-, 8.0-, 10.0-, 12.0-, 16.0-, and 24.0-h post-dose administrations in fasting conditions (periods I and II) and at 0- (pre-dose), 0.5-, 1.0-, 1.5-, 2.0-, 2.5-, 3.0-, 3.5-, 4.0-, 4.5-, 5.0-, 5.5-, 6.0-, 7.0-, 8.0-, 10.0-, 14.0- ,and 24.0-h post-dose administrations in fed state (periods III and IV). The concentration of niacin was quantitated at Lambda Therapeutic Research Ltd., Ahmedabad using LC-MS/MS validated method. The pharmacokinetic parameters were calculated from the drug concentration *vs.* time profile by the non-compartmental model using WinNonlin Professional Software Version 5.3 (Pharsight Corporation, USA) for nicotinic acid. Statistical comparison of the pharmacokinetic parameters of the two formulations was carried out using PROC MIXED of SAS® Version 9.3 (SAS Institute Inc., USA) to assess the bioequivalence of both the formulations under fasting and fed conditions.

### Pharmacokinetic (PK) analysis

Employing the estimated concentration-time profiles of niacin the following PK parameters were calculated: primary ones like c_max_, AUC_0-24h_, and AUC_0-∞_ and secondary ones like t_1/2_, t_max_ and t_lag_, as well as other PK parameters like AUC__% extrap_obs_, λ_z_. Descriptive statistics were computed and reported for all the pharmacokinetic parameters for niacin. ANOVA, two one-sided tests for bioequivalence and ratio analysis were performed on ln-transformed pharmacokinetic parameters c_max_, AUC_0-t,_ and AUC_0-∞_ for nicotinic acid under fasting (periods I and II) and fed (periods III and IV) conditions. ANOVA was also performed on untransformed pharmacokinetic parameters t_1/2_ and t_lag_, while t_max_ was analyzed using non-parametric Wilcoxon sign rank test. Bioequivalence of the test product with that of the reference product under fed and fasting conditions was concluded if the 90% confidence interval falls within the acceptance range of 80.00–125.00% for ln-transformed pharmacokinetic parameters c_max_, AUC_0-t_, and AUC_0-∞_ for nicotinic acid.

Moreover, the analysis of the plasma profiles was used to estimate how the quantity of the API that appears in systemic circulation may result in high drug plasma levels. Owing to the high variability of drug plasma levels as well as small number of subjects, the results were analyzed individually. For this purpose, drug plasma profiles and individual AUCs as areas under the plasma concentration-time curve in the timeframe 0 to 5 h (AUC_0-5h_) for fasting and 0–24 h (AUC_0-24h_) for fed conditions were calculated. The extension of the integration interval in the fed state is due to the variability of gastric emptying observed in post-prandial state for large and non-disintegrating ER tablets (26). The calculations were performed using the trapezoidal rule, and the AUCs_0-5h_ were determined by the sum of partial AUCs and the last log-linear concentration divided by the terminal disposition rate constant. Calculations of the drug amount available were performed for all subjects using equal distribution volume of 99.9 L (22).

## RESULTS AND DISCUSSION

### Standard release test using paddle apparatus and compendial dissolution media

**Fig. 1:**
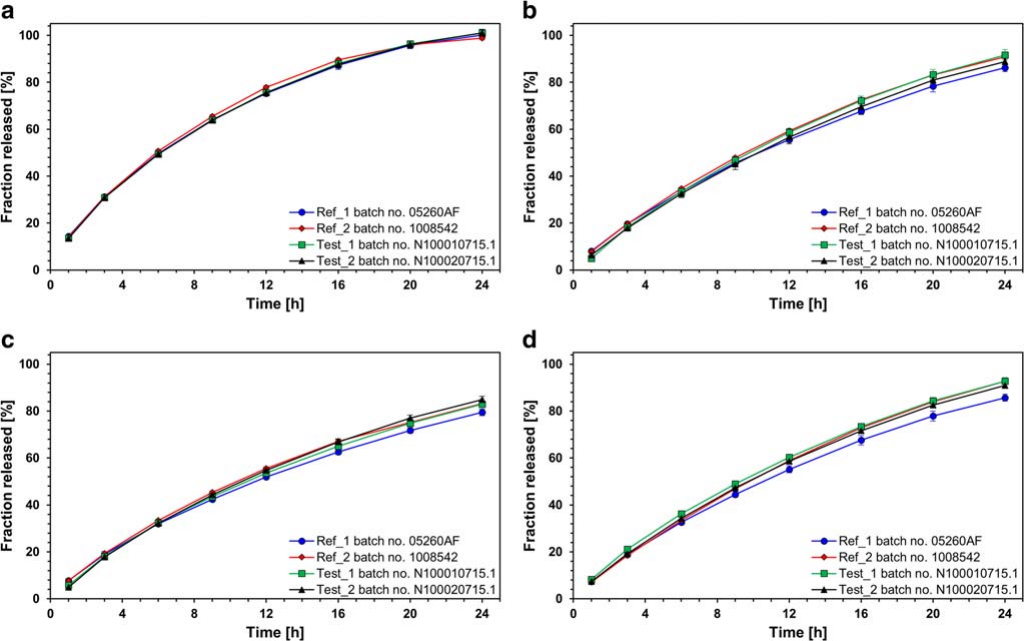
Niacin release profiles (mean ± SD, *n* = 12) from examined ER tablets obtained using paddle apparatus (900 mL, 37 ± 0.5°C, tablets placed in basket sinkers) with **a** 0.1 M HCl pH 1.2, **b** 0.05 M acetate buffer pH 4.5, **c** 0.05 M phosphate buffer pH 6.8, and **d** in purified water

Applied conditions were sufficient to release not less than 80% active substance within 24 h with acceptable relative standard deviation (RSD) at each dissolution time point (i.e., RSD < 9% at the first and < 5% in the next time points), which confirms uniform and consistent release of nicotinic acid from the hydrophilic matrices. The release of niacin from the tested ER tablets is pH dependent, with the highest release rate at pH 1.2 and the lowest at pH 6.8 (Fig. 1a and c), while release profile similarities were observed between each of examined formulations in each particular medium (f_2_ > 85 in 0.1 M HCl pH 1.2, f_2_ > 72 in 0.05 M acetate buffer pH 4.5 and in 0.05 M phosphate buffer pH 6.8, and f_2_ > 65 in purified water).

### Flow-through cell apparatus with physiologically based media pH gradients

**Fig. 2:**
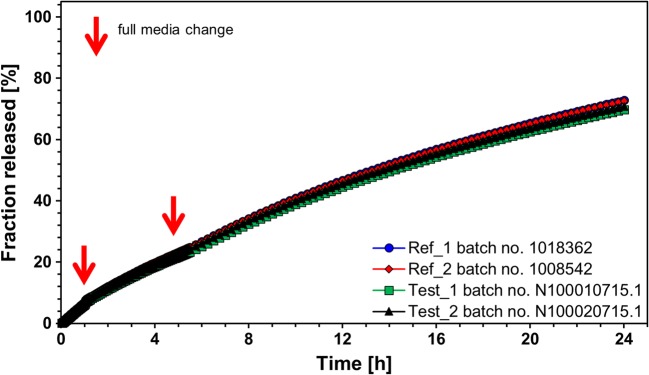
Niacin release profiles (mean ± SD, *n* = 12) from examined ER tablets obtained using flow-through cell apparatus with physiologically based media pH gradients (i.e., fasted stomach, SGFsp pH 1.8 + 0.1% Tween 80 for 1 h; fasted small intestine, blank FaSSIF pH 6.5 + 0.1% Tween 80 for 4 h; fasted colon, SCoF pH 5.8 + 0.1% Tween 80 for 19 h; flow rate 4 mL/min)

An experiment performed with the flow-through cell and applying physiological media gradients as well as transit times resulted in a moderately decreased release rate (i.e., around 70% released at 24 h, Fig. 2) compared to standard conditions (Fig. 1a–d). This observation could be explained by the slower degree of matrices hydration/swelling and their slower erosion when exposed to mild hydrodynamic conditions in the flow-through cell during “laminar-like” low flow rate at 4 mL/min. Nevertheless, in such conditions, we obtained very consistent release profiles (RSD values < 4%) and high similarity of niacin release profiles from all examined products (f_2_ > 81).

### Dynamic release stress test method under simulated fasted and fed conditions

In a recent study, it was demonstrated that the dissolution characteristic of modified release (MR) products could be remarkably influenced by physical stress events of biorelevant fortitude as they are present in human gastrointestinal tract (23). The pyloric and ileocecal regions, which are sphincters of high motor activity, were identified as the most critical stress zones of the GI tract. During the passage through these regions, dosage forms are exposed to physical stresses in the form of pressure waves with fortitude of up to 350 mbar and jet-like propulsions of chyme with peak velocities of up to 50–70 cm/s. Recently, there was established a dissolution test device which is capable of simulating physical stress conditions that are known to be present *in vivo*. Accordingly, the device can be applied for the examination of the robustness of dosage forms toward biorelevant physical stresses in order to identify the potential risk of undesired irregular release behavior *in vivo*, such as dose dumping, that may cause clinically undesired performance of the dosage forms (27). The physicochemical properties of the GI tract sections can be simulated by the utilization of GI tract-specific dissolution media. To date, it has been shown that the irregular *in vivo* release behavior of MR products observed after dosing under fasted conditions can be predicted using this novel biorelevant dissolution stress test device.

**Fig. 3:**
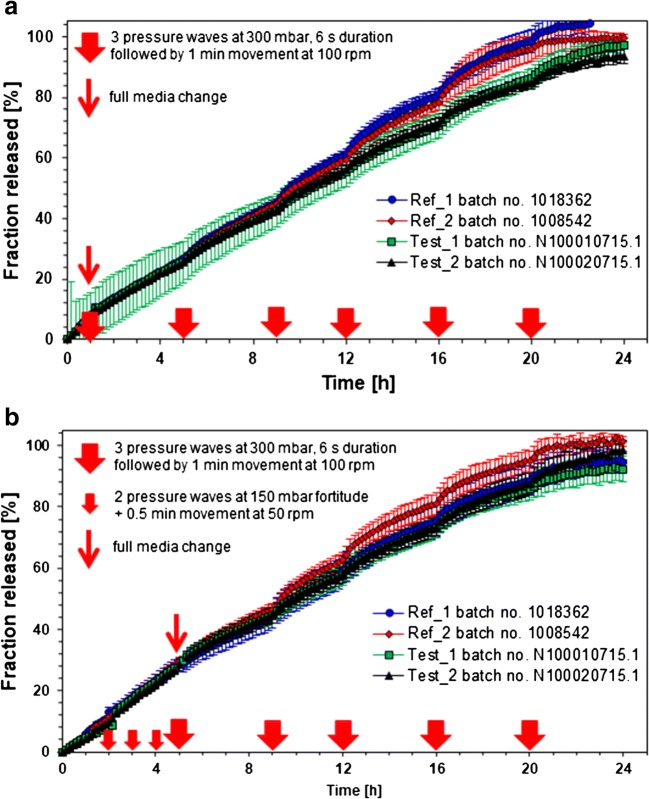
Niacin release profiles (mean ± SD, *n* = 6) from examined ER tablets obtained using dissolution stress test device under simulated fasting **a** and fed **b** conditions. Red arrows indicate the stress phases of the maximal physiological intensity, e.g., simulating the gastric emptying

The results of the *in vitro* release experiments performed under simulated fasting and fed conditions using the dissolution stress test device are presented in Fig. 3.

The media change and the mechanical stresses simulated at 1 and 5 h under the simulated fasting conditions had almost no impact on the release profiles of the tested formulations (Fig. 3a). The stress phases simulated at 9, 12, and 16 h resulted only in a slight increase in the release rates of all tested products. However, no dose dumping was observed. The tested formulations were characterized by similar release behavior over the first 12 h. After that, the release of the comparators (reference products) was somewhat faster and was completed within 20 h. The release of the drug load of the generic batches (test products) was achieved within 24 h. The released drug amount reaches values between 94 and 105% of the labeled dose. The tests yielded no meaningful interbatch differences in the release behavior of both batches of the comparator and both batches of the generic product (Table III).

**Table III:** Similarity factor (f_2_) values calculated for the batches containing 1000 mg of nicotinic acid tested under simulated fasted conditions

Product and lot number	Values of similarity factor (f_2_)			
	Niaspan, batch no. 1008542	Niaspan, batch no. 1018362	Test product, batch no. N1000010715.1	Test product, batch no. N1000020715.1
Niaspan, batch no. 1008542	–	86.23	62.83	65.44
Niaspan, batch no. 1018362	86.23	–	69.63	73.20
Test product, batch no. N1000010715.1	62.83	69.63	–	96.07
Test product, batch no. N1000020715.1	65.44	73.20	96.07	–

The media change, the intragastric conditions, and simulated gastric emptying also had almost no impact on the release of API from all the tested products under the simulated fed conditions (Fig. 3b). The stress phases simulated at 9, 12, and 16 h resulted only in a slight increase in the release rates of all the examined products. However, no dose dumping was observed. The tested formulations were characterized by similar release behavior over the entire experiment. Small differences between the reference batches were not significant. The dissolution of the drug load of all batches was achieved within 24 h. The tests yielded no meaningful interbatch differences in the release behavior of both batches of the originator and both batches of the generic product (Table IV). The dissolved drug amount ranges between 92 and 101% of the labeled dose.

**Table IV:** Similarity factor (f_2_) values calculated for the batches containing 1000 mg of nicotinic acid tested under simulated fed conditions

Product and lot number	Values of similarity factor (f_2_)			
	Niaspan, batch no. 1008542	Niaspan, batch no. 1018362	Test product, batch no. N1000010715.1	Test product, batch no. N1000020715.1
Niaspan, batch no. 1008542	**–**	72.23	87.27	90.34
Niaspan, batch no. 1018362	72.23	–	67.29	68.70
Test product, batch no. N1000010715.1	87.27	67.29	–	97.60
Test product, batch no. N1000020715.1	90.34	68.70	97.60	–

### PK analysis

**Fig. 4:**
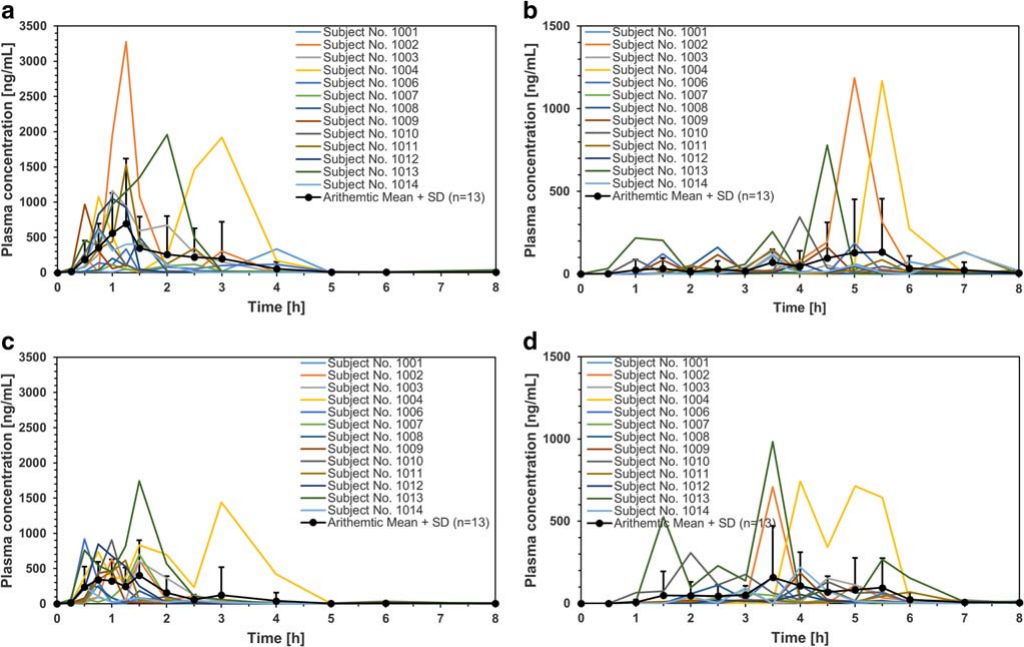
Nicotinic acid plasma profiles obtained for reference product under **a** fasted and **b** fed conditions, as well as for test product under **c** fasted and **d** fed conditions. Given are single profiles of *n* = 13 subjects as well as arithmetic mean + SD

Individual plasma nicotinic acid concentration-time profiles strongly fluctuated. Nevertheless, data from this PK study demonstrated that the test and reference products were well tolerated. There were no adverse events during the conduct of the study nor clinically significant findings in the vital signs assessment or the laboratory tests in any of the subjects in the study. The occurrence of multiple peaks in the individual plasma profiles was observed within 0.25–4 h after ingestion in the fasted state and within 1–14 h after ingestion in the fed state (Fig. 4). The occurrence of peaks cannot be temporally correlated to transport events (i.e., gastric emptying and ileocecal passage).

The AUC_0-24h_ values were 1090 ± 940 ng*h/mL and 763 ± 732 ng*h/mL, while c_max_ was 1106 ± 881 ng/mL and 743 ± 445 ng/mL for the reference and test product under fasting conditions (arithmetic means ± SD, *n* = 13), respectively. Individual t_max_ values ranged from 0.5 to 3 h (test product) or 4 h (reference product). The mean PK parameters of nicotinic acid under fed conditions were also summarized, i.e., AUC_0-24h_ values were 1140 ± 1938 ng*h/mL and 451 ± 335 ng*h/mL, c_max_ was 467 ± 607 ng/mL and 361 ± 408 ng/mL, and the individual t_max_ from 2 to 10 h and from 2.5 to 14 h for test and reference products, respectively. Based on the statistical results of 90% confidence interval for the ratio of the geometric least squares means for ln-transformed pharmacokinetic parameters c_max_, AUC_0-24h_, and AUC_0-∞_ (data not shown), conclusion was drawn for the lack of bioequivalence of test product *vs.* reference product under fasting (periods I and II) and fed conditions (periods III and IV). Significant intra- and intersubject variability for niacin under fasted and fed state was found (Table V).

**Table V:** Intra- and intersubject CV for niacin under fasting and fed conditions

Parameters	Intrasubject CV [%]		Intersubject CV [%]	
	Fasted condition (*n* = 13)	Fed conditions (*n* = 13)	Fasted condition (*n* = 13)	Fed conditions (*n* = 13)
ln c_max_	73.0	65.2	44.0	131.8
ln AUC_0-24h_	37.3	99.3	84.7	87.2
ln AUC_0-∞_	44.0	20.3	74.9	87.2

Such studies often suffer from the inability to achieve sufficient power with a low number of recruited volunteers, as demonstrated by Kosoglou et al. In their study with the aim to evaluate potential interactions of ER niacin with simvastatin/ezetimibe, 18 subjects were also too small as a sample to draw conclusions, given the fact that while geometric mean ratios for nicotinic acid c_max_ and AUC values were within 80–125% bioequivalence range, their 90% confidence intervals were unacceptably wide, e.g., 72–205% (16).

Nicotinic acid is extensively metabolized *in vivo*, including substantial but saturable first-pass metabolism that is sensitive to the rate of absorption. It results in high variability of the PK data (15,16,18). Thus, the commonly used deconvolution techniques could not be applied in our case (21,22). Therefore the suggested method of calculating partial AUCs offers a pragmatic approach and allows a simple and straightforward estimation of the amount of drug causing the occurrence of the elevated drug concentrations. The analysis of plasma profiles shows that the elevated plasma peaks occur mostly under the simulated fasted conditions in the time frame 0–5 h. The concentration peaks are, in most cases, roughly described by three to four data points only. It is likely that the occurrence of the maximal concentration was not matched with the sampling schedule. Despite the imperfections, the elevated concentration peaks deliver a major contribution to the AUC. The results are given in Fig. 4 and summarized in Table VI.

**Table VI:** Summary of the partial AUC and corresponding amounts of API calculated based on the results obtained in the clinical trial performed under fasting and fed conditions for reference (R) and test (T) products

Fasting conditions						
Subject No.	AUC_(0 - 5 h)_[(ng/mL)*h]		Drug amount in plasma (AUC 0 - 5 h) [mg]		(Drug in plasma / drug delivered at 5 h) * 100 %	
	R	T	R	T	R	T
1001	391.2	159.9	7.8	3.2	3.0	1.2
1002	2029.5	608.2	40.5	12.2	15.3	4.8
1003	1428.9	564.2	28.5	11.3	10.8	4.4
1004	2911.5	2746.3	58.2	54.9	22.0	21.5
1006	411.8	401.9	8.2	16.8	3.1	6.6
1007	207.2	439.2	4.1	8.0	1.6	3.1
1008	311.0	260.5	6.2	8.8	2.3	3.4
1009	378.6	287.2	7.6	5.2	2.9	2.0
1010	382.1	494.6	7.6	5.7	2.9	2.2
1011	1025.6	419.3	20.5	9.9	7.7	3.9
1012	761.0	680.7	15.2	8.4	5.7	3.3
1013	2505.9	1702.5	50.1	13.6	18.9	5.3
1014	571.8	193.4	50.1	13.6	18.9	5.3
Mean	1024.3	689.1	23.4	13.2	8.9	5.2
SD	912.2	730.7	19.7	13.1	7.4	5.1
Fed conditions						
Subject No.	AUC_(0 - 24 h)_ [(ng/mL)*h]		Drug amount in plasma (AUC 0 - 24 h) [mg]		(Drug in plasma / drug delivered at 5 h) * 100 %	
	R	T	R	T	R	T
1001	194.3	120.1	3.8	2.4	0.4	0.3
1002	980.5	6807.1	19.4	134.6	1.9	14.6
1003	133.3	3493.1	2.6	69.1	0.3	7.5
1004	1033.8	1366.0	20.4	27.0	2.0	2.9
1006	1687.3	256.1	33.4	5.1	3.3	0.5
1007	67.5	153.0	1.3	3.0	0.1	0.3
1008	180.0	250.0	3.6	4.9	0.4	0.5
1009	322.8	282.9	6.4	5.6	0.6	0.6
1010	518.4	570.1	10.3	11.3	1.0	1.2
1011	972.8	223.8	19.2	4.4	1.9	0.5
1012	342.6	118.5	6.8	2.3	0.7	0.3
1013	869.1	1384.1	17.2	27.4	1.7	3.0
1014	256.1	280.7	5.1	5.6	0.5	0.6
Mean	581.4	1177.3	11.5	23.3	1.2	2.5
SD	485.8	1936.8	9.6	38.3	1.0	4.2

Under the fasting conditions, the reference product provides high fluctuations in drug plasma levels. In the case of three subjects, i.e., 1002, 1004, and 1013, determined AUCs were above the threshold of 2000 [(ng/mL)*h], whereas in the case of test formulation, only in subject 1004 an AUC above 2000 [(ng/mL)*h] was achieved. Under the fed conditions, the calculated AUC was lower than in the studies performed under the fasted conditions. In the case of the reference product, the calculated AUCs were below the threshold of 2000 [(ng/mL)*h]. In the case of the test formulation only for two subjects 1002 and 1003, the calculated AUCs were significantly higher and amounted to 6807.1 and 3493.1 [(ng/mL)*h], respectively, which is mostly due to the low sampling frequency between the 12 and 24 h after the administration.

Analysis of the data obtained under the fasting and fed conditions indicates that a straightforward correlation between drug release and the apparent fraction absorbed cannot be established. Under fasting conditions, the calculated overall bioavailability of nicotinic acid in the time 0–5 h was very low and ranged for the reference product from 4.1 up to 58.2 mg (mean 23.4 ± 19.7 mg) and in the case of test formulation from 3.2 up to 54.9 mg (mean 13.2 ± 13.1 mg) for the 1000 mg dose. The calculated amount of systemically available drug corresponds up to 22% of the fraction released in the dissolution stress test device under simulated fasted conditions at 5 h.

In the case of the fed studies after excluding the subjects nos. 1002 and 1003, the calculated overall bioavailability of nicotinic acid in the time frame 0–24 h was even lower and amounted to between 6.7 and 138 mg. It corresponds to 1.3–16% of the fraction released in the dissolution stress test device under simulated fed conditions at 24 h.

**Fig. 5:**
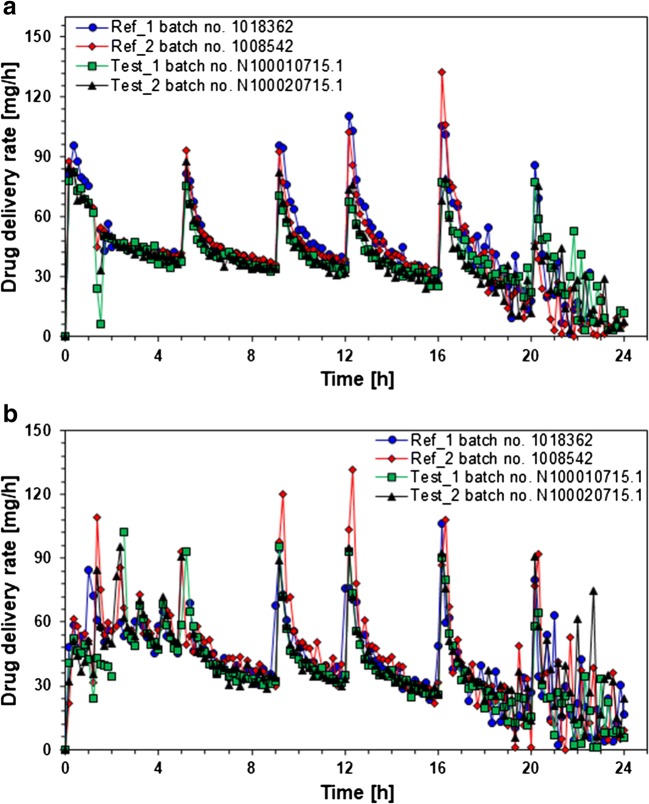
Profiles of the temporary nicotinic acid delivery rate from examined ER tablets calculated on dissolution results obtained under simulated fasted **a** and fed **b** conditions using the dissolution stress test device (given are means of *n* = 6)

The low bioavailability of nicotinic acid is due to the substantial first-pass metabolism of the drug released from ER formulations. It should be pointed out that the differences in the metabolic activity, as well as even short time increase in the drug delivery rate of ER products above approximately 40–50 mg/h upon dynamics of the GI transit conditions, may cause high fluctuations of drug plasma levels despite the low susceptibility of both examined hydrophilic matrices to physiological mechanical stress. This finding is supported by the calculations of the temporary drug delivery rate performed on the base of dissolution results obtained under simulated fasted and fed conditions using the dissolution stress test device (Fig. 5).

In the case of both products, the calculated average drug delivery rate ranges between 38 and 46 mg/h under both simulated fasted and fed conditions. However, it should be pointed out that the mechanical stresses of physiological intensity increase the temporary drug delivery rate for a short interval of 10–20 min up to 106–130 mg/h (bolus of about 16–20 mg of nicotinic acid) for the reference and up to 82–102 mg/h (bolus of up to 16 mg of nicotinic acid) for the test product, respectively. Considering the individual differences in the first-pass metabolism of nicotinic acid, it is likely that these short time spikes of the drug delivery rate may cause substantial variations of the drug plasma levels.

The lack of straightforward *in vitro–in vivo* correlation in our study despite biorelevant conditions is consistent with the findings of Kesisoglou et al. (22). While as a general rule, establishing a multiple level C IVIVC indicates the possibility of achieving a full, point-to-point level A correlation (19), this was not the case for ER niacin in the cited work. The authors developed multiple level C IVIVC between c_max_ values for both nicotinic acid and its metabolite, nicotinuric acid, and niacin amount released *in vitro* at different time points. However, traditional deconvolution approach employed to establish level A IVIVC failed to yield a positively validated model for *in vivo* prediction. Kesisoglou et al. attempted to establish a level A correlation by a customized, modified calculation method, but to little avail, which resulted only in a validated model for plasma NUA prediction, but not for the prediction of the parent compound (22).

## CONCLUSIONS

We compared two extended-release niacin products, proving their similar nicotinic acid release profile over a wide range of *in vitro* release testing conditions. The article describes the matrices’ robustness under simulated gastrointestinal mechanics and transfer conditions using a stress test device.

The paper provides evidence that erratic, multiple NA concentration peaks are a result of its local input excess over the metabolic threshold (at the level corresponding to maximum 2% of the administered dose) due to the impact of mechanical stresses of physiological intensity on examined ER tablets. We demonstrate how this behavior is similar for both marketed and test products. In this context, we describe how a properly designed and developed ER niacin test product failed to meet bioequivalence criteria against an originator product out of reasons unrelated to technology and biopharmaceutical properties. In other words, the article provides insight on how a robust extended-release matrix and well-designed formulation does not guarantee the test product’s bioequivalence to the comparator one because of the active compound’s intrinsic pharmacokinetic characteristics, i.e., highly variable, extensive metabolism of nicotinic acid.

## Electronic supplementary material

ESM 1 (DOCX 1.02 mb)
