# Supplementary material for: Biorelevant In Vitro Release Testing and In Vivo Study of Extended-Release Niacin Hydrophilic Matrix Tablets
Source: AAPS PharmSciTech. 2020 Jan 27;21(3):83. doi: 10.1208/s12249-019-1600-z (PMC6985042; doi:10.1208/s12249-019-1600-z)
Supplement: Supplementary file 1 — (DOCX 1.02 mb) [file 12249_2019_1600_MOESM1_ESM.docx]

**Supplementary Material**

**Table SI.** USP 4 release test conditions.

| **Parameter** | **Set** | | | | |
| --- | --- | --- | --- | --- | --- |
|  |  |  |  |  |  |
| Flow-Through Apparatus Configuration | Open on-line loop system with media selector | | | | |
| Medium temperature [°C] | 36.967 ± 0.037 | | | | |
| Flow rate [mL/min] | 4.00 ± 0.04 | | | | |
| Flow type | “Laminar”: Packed Column | | | | |
| Total time of analysis [h] | 24 | | | | |
| Tablet positioning | On tablet holder | | | | |
| Video monitoring | Pictures were taken every 1h from 0 to 24h (25 jpg pictures per run) | | | | |
| Dissolution medium | 0.1% Tween^®^ 80 in Simulated Gastric Fluid (SGF) pH=1.8 (0-1h) | | | | |
|  | 0.1% Tween^®^ 80 in blank Fasted State Simulated Intestinal Fluid (FaSSIF blank) pH=6.5 (1-5h) | | | | |
|  | 0.1% Tween^®^ 80 in Simulated Colonic Fluid (SCoF) pH=5.8 (5-24h) | | | | |
| Medium volume per cell [L] | 0.24 L (0.1% Tween^®^ 80 in SGF pH=1.8) | | | | |
|  | 0.96 L (0.1% Tween^®^ 80 in FaSSIF blank pH=6.5) | | | | |
|  | 4.56 L (0.1% Tween^®^ 80 in SCoF pH=5.8) | | | | |
| Niacin determination | UV on-line spectrophotometry | | | | |
| Detection wavelength λ [nm] | 262 | | | | |
| Cuvettes pathlength [mm] | 1 | | | | |
| Blank | Dissolution medium | | | | |
| Quantification | Based on niacin E11* value in various dissolution media | | | | |
|  | 0.1% Tween^®^ 80 in SGF pH=1.8 | | 0.1% Tween^®^ 80 in FaSSIF blank pH=6.5 | | 0.1% Tween^®^ 80 in SCoF pH=5.8 |
|  | E11 = 396.430 (n=9, RSD=1.05%) | | E11 = 253.729  (n=9, RSD=1.08%) | | E11 = 262.575 (n=9, RSD=1.26%) |
|  | Linearity | | | | |
|  | 150 – 350 mg/L (r = 0.99997) | | 50 – 250 mg/L  (r = 0.99995)  250 – 1250 mg/L (r = 0.99997) | | 25 – 200 mg/L  (r = 0.99996) |
| Sampling | Samples | Interval [min] | Test time [min] | Medium | |
|  | 25 | 2 | 50 | 0.1% Tween^®^ 80 in SGF pH=1.8 | |
|  | 10 | 1 | 10 | 0.1% Tween^®^ 80 in SGF pH=1.8 | |
|  | 15 | 1 | 15 | 0.1% Tween^®^ 80 in FaSSIF blank pH=6.5 | |
|  | 43 | 5 | 215 | 0.1% Tween^®^ 80 in FaSSIF blank pH=6.5 | |
|  | 10 | 1 | 10 | 0.1% Tween^®^ 80 in FaSSIF blank pH=6.5 | |
|  | 30 | 1 | 30 | 0.1% Tween^®^ 80 in SCoF pH=5.8 | |
|  | 74 | 15 | 1110 | 0.1% Tween^®^ 80 in SCoF pH=5.8 | |

* The E11 value is automatically calculated by WinSOTAX Plus Dissolution software and it corresponds to the grade of the regression, which can be drawn through the data points on a plot of the measured absorbance’s against the standard concentrations.

The regression grade (B) is given by:

The total value of E11 is given by:

***Dissolution stress test device***

The apparatus aims at simulating the dimensions of physiological mechanical stress that may occur during the GI tract passage of a solid dosage form. For this objective, the dissolution stress test apparatus exposes a dosage form to sequences of agitation including movement and pressure fluctuations alternated with static phases as observed in vivo. Moreover, the device enables the simulation of an intermittent contact of the dosage form with the dissolution medium. The device is illustrated in Fig. S1, and a more detailed description can be taken from references (1, 2).


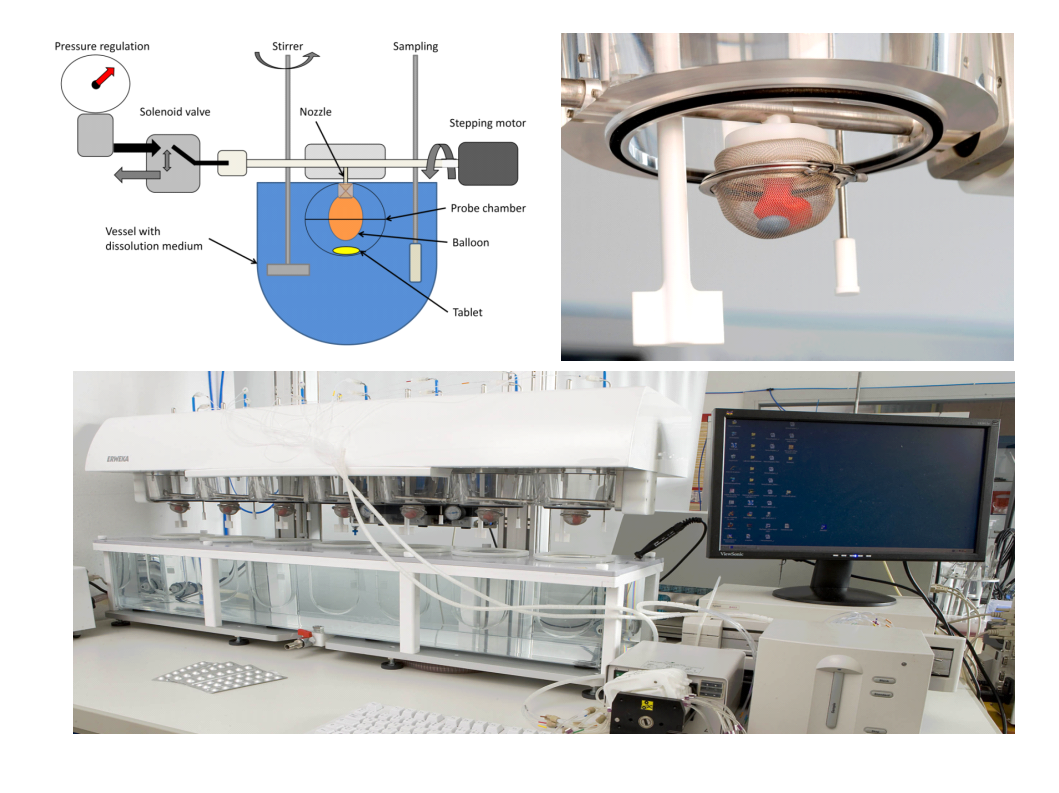


**Fig. S1.** Bio-relevant dissolution stress test device: schematic representation, construction detail and photographic representation of the test setup with flow-through UV Vis spectrophotometer (2).

***Blood samples preparation***

The pre-dose blood samples (7 ml each) were collected within 60 minutes before the dosing. Post-dose samples (5mL each) were collected with an acceptable deviation of 2 minutes from the scheduled time for all samples. Immediately after each tube of blood is drawn , it was inverted several times gently to ensure the mixing of tube contents (i.e., anticoagulant). The samples were centrifuged at 3000 ± 100 rcf for 5 minutes below 10 °C to separate plasma. The blood samples were kept in an ice-cold water bath before centrifugation and during separation. The separated plasma samples were transferred to pre-labeled polypropylene tubes in two aliquots, i.e., around 0.7 mL in the first aliquot (around 1.2 mL in case of pre-dose samples) and rest of the volume in the second aliquot as a backup lot. All the samples were stored upright in a box containing dry ice or in a freezer at temperature -65 ± 10° C for interim storage if required. Finally, the samples were transferred by keeping them in the dry ice box and stored in a bioanalytical freezer at -65 ± 10° C until completion of analysis.

***PK analysis***

Semilogarithmic plots of individual and mean plasma concentration versus time profiles from the *in vivo* study for the reference and test ER formulations under fasting and fed conditions are presented in Fig. S2.


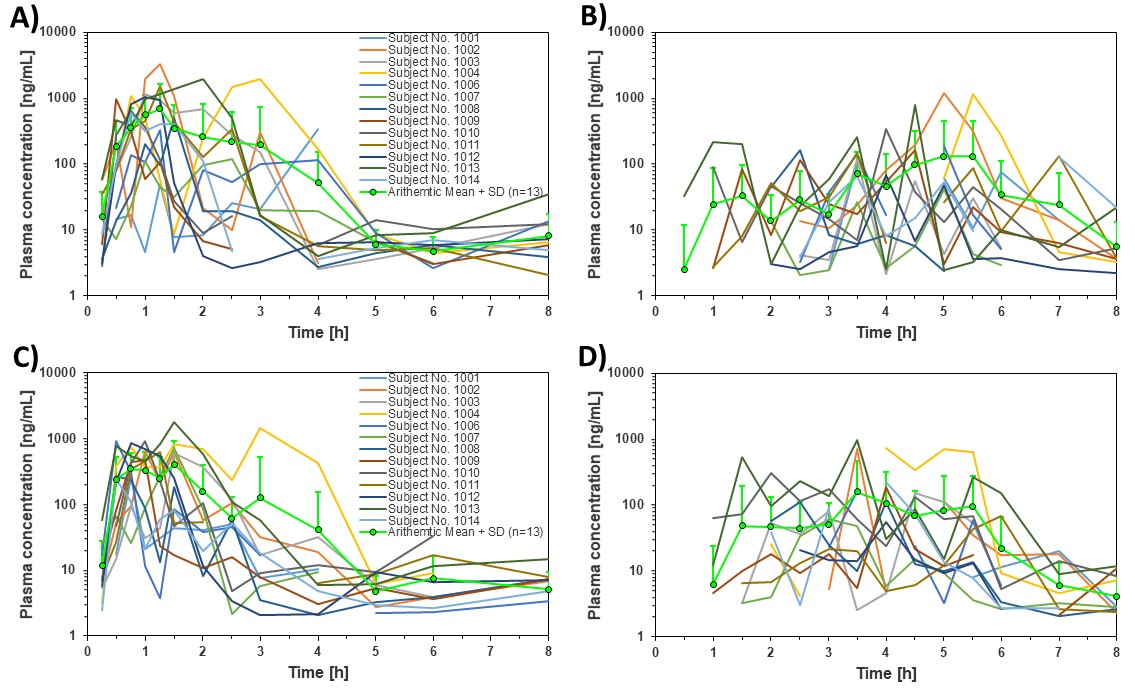


**Fig. S2.** Log-linear scale of individual and mean (n = 13) nicotinic acid plasma concentration versus time profiles obtained for reference product under **(A)** fasted and **(B)** fed conditions, as well as for test product under **(C)** fasted and **(D)** fed conditions.

**References**

1. G. Garbacz, R.-S. Wedemeyer, S. Nagel, T. Giessmann, H. Mönnikes, C.G. Wilson, W. Siegmund, W. Weitschies, Irregular absorption profiles observed from diclofenac extended release tablets can be predicted using a dissolution test apparatus that mimics in vivo physical stresses, Eur. J. Pharm. Biopharm. 2008;70:421–8. doi:10.1016/j.ejpb.2008.05.029.
2. G. Garbacz, S. Klein, W. Weitschies, A biorelevant dissolution stress test device - background and experiences, Expert Opin. Drug Deliv. 2010;7:1251–61. doi:10.1517/17425247.2010.527943.
